# Impact of Gentamicin-Loaded Bone Graft on Defect Healing in a Sheep Model

**DOI:** 10.3390/ma12071116

**Published:** 2019-04-04

**Authors:** Elisabeth Beuttel, Nicole Bormann, Anne-Marie Pobloth, Georg N. Duda, Britt Wildemann

**Affiliations:** 1 Julius Wolff Institute and Berlin-Brandenburg Center for Regenerative Therapies, Charité-Universitätmedizin Berlin, Corporate Member of Freie Universität Berlin, Humboldt-Universität zu Berlin, and Berlin Institute of Health, 13353 Berlin, Germany; elisabeth.beuttel@posteo.de (E.B.); nicole.bormann@charite.de (N.B.); anne-marie.Pobloth@charite.de (A.-M.P.); georg.duda@charite.de (G.N.D.); 2 Experimental Trauma Surgery, University Hospital Jena, 07740 Jena, Germany

**Keywords:** bone infection, local drug delivery, bone graft, demineralized bone matrix, gentamicin, regeneration

## Abstract

Infections of bone are severe complications, and an optimization of grafting material with antimicrobial drugs might be useful for prevention and treatment. This study aimed to investigate the influence of gentamicin-loaded bone graft on the healing of bone defects in a sheep model. Metaphyseal and diaphyseal drill hole defects (diameter: 6 mm, depth: 15 mm) were filled with graft or gentamicin-loaded graft (50 mg/g graft) or were left untreated. Analysis of regeneration after three and nine weeks, micro-computed tomography (μCT), and histology revealed a significant increase in bone formation in the drill hole defects, which began at the edges of the holes and grew over time into the defect center. The amount of graft decreased over time due to active resorption by osteoclasts, while osteoblasts formed new bone. No difference between the groups was seen after three weeks. After nine weeks, significantly less mineralized tissue was formed in the gentamicin-loaded graft group. Signs of inflammatory reactions were seen in all three groups. Even though the applied gentamicin concentration was based on the concentration of gentamicin mixed with cement, the healing process was impaired. When using local gentamicin, a dose-dependent, compromising effect on bone healing should be considered.

## 1. Introduction

Infection of bone is a devastating complication for the patient. If the infection cannot be resolved by systemic antibiotic treatment, surgery with debridement of all infected tissue is necessary [1]. This radical debridement often results in larger bone defects that require support to allow the regeneration of the bone. The treatment of this large bone defects has consequently two challenges: 1. the regeneration of the defect, 2. the continuous prophylaxis or lasting treatment of infections. To support the regeneration of bone, autologous bone is the gold standard, but has limitations such as harvest-side morbidity, and sometimes limited availability should be carefully considered [2,3]. Allografts, xenografts, alloplastic materials, regeneration stimulating factors such as BMP-2, and a combination thereof are clinically used and new materials are under development [4,5]. Sometimes, such materials are combined with antibiotics to prevent the onset of infection. The first antibiotic-mixed bone cement for local drug delivery was described 1970 by Buchholz and Engelbrecht [6]. Since then such bone cements have been commonly used in arthroplasty and the effectiveness of antibiotic impregnated nondegradable cements to reduce the infection rate in primary hip arthroplasty was shown [7]. In recent decades, various approaches for the local delivery of antibiotics for prevention and treatment of bone infections were developed [8,9]. Clinically used is the direct application of powdered antibiotics during orthopedic surgery [10], but also antibiotics mixed with allografts [11], applied in combination with degradable bioceramics [12], or from implant coatings [13]. Gentamicin, however, is not only toxic for bacteria, it can have—above certain concentrations—negative effects on bone-forming cells [14]. Thus, for successful healing, it is important that the antibiotic dosage eradicates bacteria but does not impair bone regeneration. To this end, loading of grafting material with antibiotics for direct delivery to the site of need without harming the endogenous healing process is desired. In a previous study, we developed and tested a perioperative loading method of a bone graft with antibiotics [15]. Incorporated gentamicin showed a first-order release with almost complete release within the first week. Eluates from this period were antimicrobial active against *S. aureus*, without cytotoxic effects on primary osteoblast-like cells. The present study aimed to investigate the effect of this local gentamicin application on the regeneration of bone defects. A large animal model was used, and drill holes in the epiphyseal and metaphyseal bones were filled with grafting material with and without gentamicin enrichment. After three and nine weeks, the healing was assessed using micro-computed tomography (μCT) and histology. 

## 2. Materials and Methods

The bone graft demineralized bone matrix (DBMputty, DIZG, Berlin, Germany) was mixed with gentamicin (40 mg/mL, Merckle GmbH, Blaubeuren, Germany) using a syringe with an integrated mixing propeller (Medmix Systems, Risch-Rotkreuz, Switzerland) [15]. 

### 2.1. Drill Hole Defect Model

For in vivo analysis, drill hole defects were created in diaphyseal and metaphyseal bones of five sheep. Defects (6–8 per group and time point) were filled with demineralized bone matrix (DBM) or DBM loaded with gentamicin (50 mg/g DBM, same concentration as in the *in vitro* studies [15]) or left untreated. The drill holes in all animals were assigned to the three experimental groups. After three and nine weeks, defect regeneration was analyzed by µCT and histology. This study was part of a larger study and described in more detail in [16,17].

The animal experiment followed national and international regulations for the care and use of laboratory animals and was approved by the local authorities (LaGeSo, Berlin, Approval No. G0341/12). This study was part of a larger study and in total 16 adult female merino-mix sheep (mean weight 65 kg; ±7 kg; age 2.5 years) were included. Drill holes (diameter 6 mm and depth max. 15 mm) were drilled in the metaphyseal part of femur and humerus and the diaphyseal part of metacarpus and metatarsus and filled with 200 mg of DBM with or without 10 mg gentamicin. Empty defects served as controls. In the first surgery, defects were drilled and filled in the right extremities and after six weeks holes were drilled and filled in the left extremities. Animals were sacrificed after further three weeks. Therefore, healing times were three and nine weeks. 

### 2.2. Surgery

The grafting material was rehydrated immediately prior to surgery. The surgery was conducted under general anesthesia introduced by intravenous sedation (10–16 mg/kg body weight sodium thiopental; Trapanal^®^, Nycomed Deutschland GmbH, Konstanz, Germany) followed by endotracheal inhalative anesthesia (2% isoflurane, 12–20 breath/min) accompanied by intravenous infusion, intraruminal tubation, analgetic treatment and gastritis and infection prophylaxis (amoxillin and metronidazole). To ensure a standardized placement of the drill holes in the individual sheep the localizations were marked preoperatively using radiographic surgical imaging. Following sterile preparation, the bone localizations were prepared surgically, and the drill holes were placed in a standardized fashion using a custom-made template and a surgical drill (Arthrex^®^ 6.0 mm drill bit, Arthrex, Munich, Germany). For a more detailed description of the operation, pain management, and animal care see previous publication [16]. Each defect was filled completely with the bone graft using the preparations syringe or left empty followed by a surgical wound closure. Animals were sacrificed after 9 weeks by sedation with 25–30 mg/kg sodium thiopental intravenously and application of 100 mL potassium chloride.

### 2.3. Serum Concentration of Gentamicin

In total 30 blood samples were taken preoperative, at day 1 and 5 post surgery of all five sheep (two operations per sheep). The gentamicin concentration was determined by the Labor Berlin (Charité Vivantes, Berlin, Germany) using KIMS method (kinetic interaction of microparticles in a solution; Roche, Germany).

### 2.4. Micro-Computed Tomography (µCT)

After collection, bone samples were digitally imagined using X-ray to exclude ectopic bone formations and scanned in the µCT (VivaCT 40, SCANCO medical AG, Brüttisellen, Switzerland). The volume of interest (VOI) was defined as three-dimensional cylinder of the drill holes. The percentage of bone volume/total volume (BV/TV) was determined via a threshold based on Hounsfield Units (HU), which were calculated for each sample based on adjacent intact bone regions (mean 189.9 HU). 

### 2.5. Histology and Histomorphometry

Undecalcified bone samples were embedded in methyl methacrylate (Technovit 9100 NEU, Kulzer GmbH, Wehrheim, Germany). Sections (6 µm) were stained with Safranin Orange/von Kossa (black: mineralized tissue, red: soft tissue) and Movat Pentachrome (yellow: collagen fibers; light blue: reticular fibers; red: elastin fibers). Active osteoclasts were stained using TRAP (Tartrate Resistant Acid Phosphatase) and Methyl Green as counter staining. For histomorphometry, bone tissue, DBM, connective tissue, and the percentage of osteoblasts and osteoclasts covering the DBM surface were quantified using ImageJ 1.49 (NIH, Bethesda, MD, USA). 

### 2.6. Statistical Analysis

For statistical analysis of the differences between the three groups Kruskal-Wallis test followed by Dunn’s was performed (GraphPad Prism 8, San Diego, CA, USA). Differences within one group between the two time points was assessed with the Mann-Whitney-U Test. Values *p* ≤ 0.05 were considered as significant.

## 3. Results

Experimental design followed the 3R principles for animal models to screen for treatment effects while reducing the number of animals necessary. Therefor control groups (control and DBM) were used also in another study and the results are partially published [17].

### 3.1. µCT Analysis of the Defect Healing

**Figure 1:**
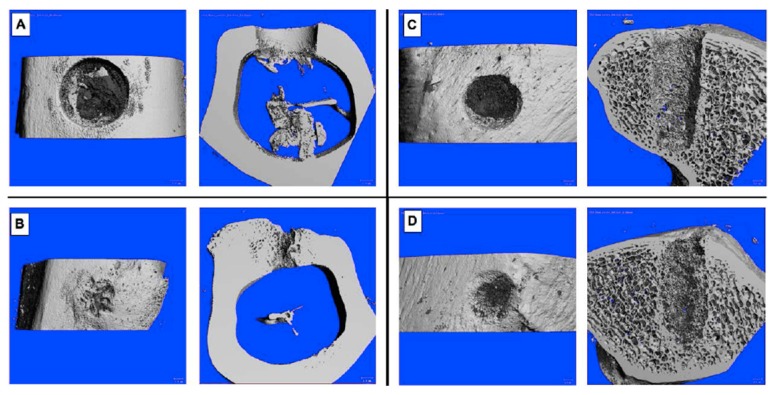
Exemplary drill hole defects after three (**A**,**C**) and nine weeks (**B**,**D**). (**A**,**B**) diaphyseal defects, filled with gentamicin-loaded DBM, (**C**,**D**) metaphyseal defects, filled with DBM.

**Figure 2:**
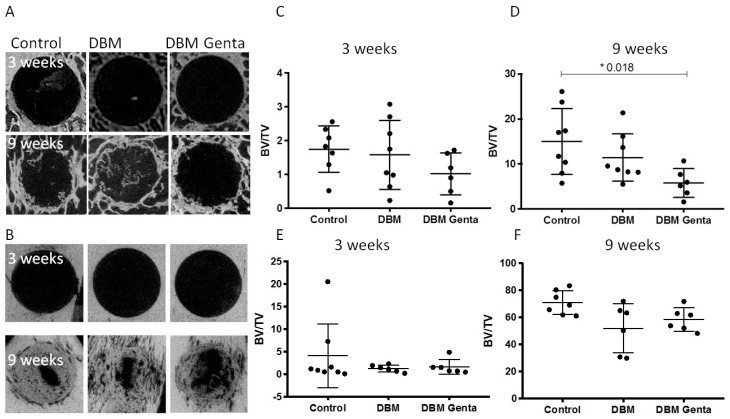
µCT images of representative metaphyseal and diaphyseal bone defects after three and nine weeks of healing (**A**,**B**). The metaphyseal defects (**A**) showed healing only at the edges of the defect, whereas the diaphyseal defects (**B**) showed a progressed healing after nine weeks. (**C**–**F**) Quantification of the BV/TV in the defects at three and nine weeks in metaphyseal and diaphyseal bone. The data from the control and DBM treated metaphyseal defects were published previously [17]. A significantly reduced BV/TV was seen after nine weeks in the metaphyseal drill holes treated with gentamicin. Kruskal-Wallis test with Dunn´s (n = 6–8 per group and time point).

The µCT reconstructions showed only limited new bone formation in the diaphyseal and metaphyseal defects after three weeks independent of the treatment (Figure 1). After nine weeks of healing, a clear difference between the diaphyseal and metaphyseal defects was visible, with an increased healing in all diaphyseal defects (Figure 1 and Figure 2).

Quantification of the newly formed bone by µCT showed significantly less bone formation in the gentamicin-treated metaphyseal defects after nine weeks compared to control defects (Figure 2D). The amount of newly formed bone increased significantly in all three groups from week three to week nine in the diaphyseal as well as in the metaphyseal drill hole defects (*p* < 0.009). 

### 3.2. Histological Analysis of the Defect Healing

**Figure 3:**
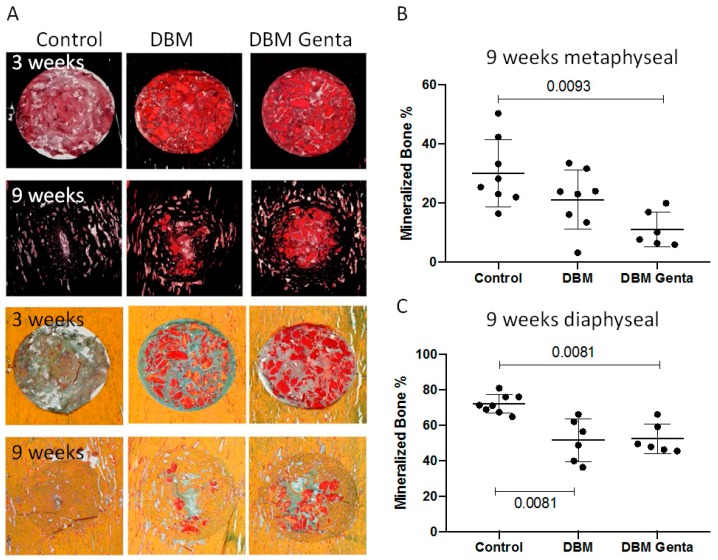
Exemplary histological pictures of the diaphyseal drill hole defects. Tissue was stained with Safranin O/van Kossa (**A**, top) to visualize the mineralized bone, or with Movat Pentachrome (**A**, bottom) to differentiate the tissues. Healing progressed from week three to week nine in all groups, with less healing in the gentamicin-treated defects. (**B**,**C**) Histomorphometric analysis: After nine weeks, significantly less mineralized tissue was formed in the gentamicin-loaded DBM group, both metaphyseal and diaphyseal. Diaphyseally, significantly less bone was formed in the DBM group compared to control. Kruskal-Wallis test with Dunn´s (n = 6–8 per group and time point).

The histological evaluation revealed new bone formation starting at the edges of the drill holes and growing into the center over time (Figure 3A). The histomorphometric analysis showed no significant differences in the tissue composition after 3 weeks. After nine weeks, significantly less mineralized tissue was quantified in the gentamicin-treated defects compared to the control defect. This was independent from the localization (Figure 3B,C). In the diaphyseal defects significantly less mineralized tissue was formed in the DBM group compared to the control defects (Figure 3C). 

**Figure 4:**
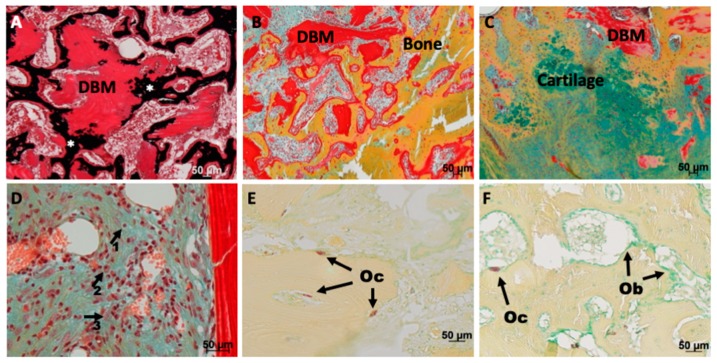
Safranin O/van Kossa (**A**), Movat Pentachtome stain (**B**–**D**) or TRAP stain (**E**,**F**) of drill hole defects after three (**A**,**B**,**D**,**E**) or nine (**C**,**F**) weeks healing. (**A**) Remineralization (black, *) of the implanted DBM (intense red stained tissue with empty lacunae) occurred after three weeks. (**B**) Active remodeling of the DBM was visible: DBM was attached to and integrated into newly formed bone trabeculae. (**C**) Endochondral bone formation was only seen in a few drill holes filled with DBM. (**D**) No difference regarding the abundance of inflammatory cells was detectable between the groups; lymphocytes (arrow 1), macrophages (arrow 2) or histiocytes (arrow 3) were detectable in all groups. (**E**) Osteoclasts (Oc) were detectable on the surface of the DBM, but also inside DBM after three weeks. F) Shift to the coverage with mostly osteoblasts (Ob) compared to osteoclasts (Oc) at the late time point. Size of scale bars is given in the figures.

Comparing the changes over time within one group revealed in all three groups a significant increase in the amount of mineralized bone from week three to week nine in both localizations (*p* < 0.026). The amount of DBM decreased over time with a significantly lower amounts after nine weeks in the DBM group (*p* < 0.009). The remodeling in the gentamicin-loaded DBM group was less pronounced with no significant reduction. The remodeling process can be explained by the activity of osteoclast and osteoblasts. A shift from resorption by osteoclasts (TRAP stain) at three weeks after implantation to formation of new bone by osteoblast after nine weeks was visible (Figure 4E,F). Counting the cells, however, revealed no significant differences between the groups (data not shown). Interestingly, the DBM was not only remodeled at the surface, but also osteoclasts were seen in the DBM (Figure 4E). Mineralized tissue connected to DBM was visible, but also a remineralization of the DBM (Figure 4A,B). Bone formation in most of the defects was due to direct ossification and only a few small cartilage islands were seen in the DBM treated defects (Figure 4C). At the cellular level, no increased inflammatory reaction was seen in the DBM treated groups. Independent of the group, defects after three weeks of healing contained leukocytes, macrophages and other cells of the immune system (Figure 4D). 

### 3.3. Serumconcentration of Gentamicin

In 23 blood samples the concentration of gentamicin was below the lower detection level and in seven samples of two sheep in a range between 0.55–0.73 mg/L.

## 4. Discussion

Local antibiotic therapies are frequently used to prevent or treat bone infection. They can either be applied via loading of cements or grafting materials or by direct application into the defect area. Beside infection prophylaxis or treatment, the effect on bone regeneration is important. This study used gentamicin-loaded resorbable bone graft, which was characterized in detail in a previous study [15], to treat defects in sheep bone. First signs of bone formation were seen in all three groups after three weeks of healing without differences. In the following six weeks the gentamicin-treated defects showed impaired healing resulting in significantly less bone as shown by µCT and histological analysis. This healing impairment after 9 weeks was unexpected, as no impairment was seen after 3 weeks, the time period when all gentamicin should be released. In addition, no negative effect on osteoblast-like cells was seen in the *in vitro* characterization study using the same ratio of gentamicin and DBM: 50 mg/g DBM [15]. The previous *in vitro* experiments revealed a burst and almost complete release of gentamicin after three days, antimicrobial activity and good cytocompatibility of the eluates. The metabolic activity decreased only with the 1-day eluates, but without significant difference, whereas alkaline phosphate activity was not affected [15]. High gentamicin concentration was used for the *in vitro* experiments: 10 mg gentamicin mixed with 200 mg graft resulting in 2 mg gentamicin/mL medium. Previous studies showed that concentrations of 100 and 200 µg/mL reduced the DNA content but had no effect on viability of marrow derived mesenchymal stem cells [18]. Interestingly, the effect of gentamicin exposure was transient and affected cell proliferation and alkaline phosphatase activity only after 4 and 8 days of initial incubation but not after longer cultivation without gentamicin. The gentamicin/DBM concentration chosen for the presented experiments was within the range of clinically used antibiotic-loaded cement for prophylaxis and treatment in joint replacement [19]. We are not aware of a study reporting impaired healing due to the gentamicin released from the cements. The release from the DBM, however, is much faster and almost complete after 3 days *in vitro*, whereas gentamicin is released from cements over years [20]. This results in a much higher local concentration when using DBM as a carrier instead of cement. Using allografts, attenuated healing was also seen in spinal fusion where DBM was mixed with vancomycin [21], whereas DBM with gentamicin or DBM/bioactive glass with tobramycin had no negative effect on osteoinductivity or femur defect healing, respectively [22,23]. Coraca-Huber et al. published detailed studies investigating the effect of e.g., processing, storage, and freezing on antibiotic-loaded allogeneic bone and found promising *in vitro* results; however, no in vivo study has been published investigating biocompatibility until now [11,24,25,26,27]. 

No negative effect of calcium sulfate impregnated with gentamicin was seen in vivo [28]. The material was implanted into intact rabbit bone and material resorption as well as bone formation was monitored over 12 weeks. The amount of gentamicin loaded to the substitute was 1.7% compared to 5% used in the present study. This lower dosage might explain the contradictory results, although the different models—bone formation vs. defect healing—should be considered. The slowly resorbing material was still visible after 12 weeks and surrounded by giant cells. In a previous study the group showed a release of 82% of the gentamicin within three days [29], which is similar to the release of gentamicin from the bone graft used in the present study. Using the same material, no negative effect on blood coagulation was seen [30]. 

Gentamicin was not detectable in the serum in most of the samples and only at low levels in 7 of 30 samples (0.55–0.73 mg/L). Calculating the theoretical gentamicin concentration based on the amount loaded to the DBM, a concentration between 4.4 mg/L and 8.7 mg/L in the serum of the sheep might have been possible. Due to the release kinetics and the half-life of gentamicin these concentrations were not expected systemically, and the low or undetectable systemic concentration is an important safety aspect for the use as a local antibiotic application with the advantage of reducing possible systemic side effects to a minimum.

In the present study an ovine drill hole model was used that was developed for the screening of bone tissue engineering strategies [16]. The advantage of this model is a similar bone remodeling as in human [31], the standardization of the defects and the investigation of several defects in one animal. This is in line with the 3R principals and results in a reduction of the needed animals. Limitations are the certain size of the drill holes and the plexiform bone of sheep with less harversian canals [31]. Dogs, goats, pigs, or rabbits might be alternative animal models, but they have all their advantages and disadvantages and do not exactly mirror the human situation.

Taken together, even after *in vitro* testing showing good cytocompatibility in the cell culture experiment, the gentamicin-loaded graft impaired new bone formation in the current sheep model. Similar concentrations of gentamicin are used in combination with cement; the fast release of gentamicin from the graft, however, results in higher local concentrations in vivo.

## 5. Conclusions

Local application of antibiotics is often used in the treatment of bone defects for prophylactic or therapeutic purposes. High concentrations of antibiotics, however, may impair bone regeneration, potentially due to cytotoxic effects.
